# Addressing microaggressions in racially charged patient-provider interactions: a pilot randomized trial

**DOI:** 10.1186/s12909-020-02004-9

**Published:** 2020-03-24

**Authors:** Jonathan W. Kanter, Daniel C. Rosen, Katherine E. Manbeck, Heather M. L. Branstetter, Adam M. Kuczynski, Mariah D. Corey, Daniel W. M. Maitland, Monnica T. Williams

**Affiliations:** 1 University of Washington, Seattle, USA; 2 Bastyr University, Kenmore, USA; 3 Texas A&M University Corpus Christi, Corpus Christi, USA; 4 University of Connecticut, Mansfield, USA

## Abstract

**Background:**

Racial bias in medical care is a significant public health issue, with increased focus on microaggressions and the quality of patient-provider interactions. Innovations in training interventions are needed to decrease microaggressions and improve provider communication and rapport with patients of color during medical encounters.

**Methods:**

This paper presents a pilot randomized trial of an innovative clinical workshop that employed a theoretical model from social and contextual behavioral sciences. The intervention specifically aimed to decrease providers’ likelihood of expressing biases and negative stereotypes when interacting with patients of color in racially charged moments, such as when patients discuss past incidents of discrimination. Workshop exercises were informed by research on the importance of mindfulness and interracial contact involving reciprocal exchanges of vulnerability and responsiveness. Twenty-five medical student and recent graduate participants were randomized to a workshop intervention or no intervention. Outcomes were measured via provider self-report and observed changes in targeted provider behaviors. Specifically, two independent, blind teams of coders assessed provider *emotional rapport* and *responsiveness* during simulated interracial patient encounters with standardized Black patients who presented specific racial challenges to participants.

**Results:**

Greater improvements in observed emotional rapport and responsiveness (indexing fewer microaggressions), improved self-reported explicit attitudes toward minoritized groups, and improved self-reported working alliance and closeness with the Black standardized patients were observed and reported by intervention participants.

**Conclusions:**

Medical providers may be more likely to exhibit bias with patients of color in specific racially charged moments during medical encounters. This small-sample pilot study suggests that interventions that directly intervene to help providers improve responding in these moments by incorporating mindfulness and interracial contact may be beneficial in reducing racial health disparities.

## Background

Disparities in medical care and outcomes for Black patients compared to White patients in the US are well documented [1] and have not changed significantly for decades [2]. Among the multiple factors responsible for these disparities [3, 4], substantial evidence exists that providers themselves contribute to the problem through biased provider-patient interactions [5, 6]. Many of these biases expressed in provider-patient interactions are seen as operating on implicit levels, in that the biases that influence behavior are activated automatically and outside the provider’s awareness, are difficult for the provider to control, and occur in contrast to the provider’s explicitly espoused anti-racist attitudes and identity [5, 6]. For example, White providers who score higher on measures of implicit bias, but not explicit bias, speak faster, dominate conversations, have shorter visits [7, 8], display fewer positive nonverbal cues [9] and less warmth [10], and use more first-person plural pronouns and anxiety-related words [11, 12] when interacting with Black patients. In turn, both Black patients and independent observers provide lower ratings of visit satisfaction and patient-centered care for White providers [7, 10, 13, 14].

Of particular concern is the effect of bias on providers’ capacity to express emotional rapport. Emotional rapport, variously defined as displaying empathy, respect, validation, and concern for the patient [15–17], is central to patient-centered care, independently predicts Black patients’ trust in providers [18], and may be particularly sensitive to the effects of bias. For example, Black-White disparities in the quality of provider’s emotional rapport have been well documented, including in general primary care settings [14], and when patients present with uncontrolled hypertension [19] or depression [20]. Emotional rapport may be particularly challenging for providers to establish when negative stereotypes and implicit biases are activated by certain normative Black presentations [21]. This may be alarmingly common, such as whenever issues of socio-economic status or race are made salient in the encounter [22] or when a patient discloses past incidents of discrimination. In these racially charged moments, a provider’s biased responses may include shifts in attention to focus on the racial features of the other person (e.g. [23]), expressions of automatic, inaccurate, negative stereotypes [21, 22], heightened physiological threat responses [24] and anxiety [25, 26], and avoidance of discussions of race and racism [27].

Such responses are experienced as microaggressions by many Black patients. Microaggressions, first coined by Pierce [28], are most often defined as brief, everyday, intentional or unintentional verbal and non-verbal behavioral expressions that communicate hostile, derogatory, or negative racial slights and insults to the oppressed target person or group [29]. Microaggressions are experienced frequently by Black people and other people of color, including in health care settings [27, 30], and an increasing number of studies document that these experiences predict distress, distrust, and disengagement in multiple samples [31, 32]. Furthermore, in response to a White provider’s observable interracial anxiety and other microaggressive behavior, a Black patient may experience distress and understandably disengage from the encounter given past personal negative experiences and historical medical abuses (e.g., Tuskegee), further (and paradoxically) activating provider biases and inaccurate stereotypes of non-compliance [33, 34].

Despite the importance of addressing harmful expressions of bias in medical encounters, evidence for the effectiveness of interventions that focus directly on decreasing such bias is mixed, with some evidence for changes in measures of implicit bias but no evidence for corresponding changes in provider behavior [35]. Furthermore, although there is increasing interest in intervening on microaggressions in healthcare [36], to date no such interventions have been empirically evaluated. Innovations are needed.

The goal of the present study was to test a brief training intervention designed to decrease the likelihood of microaggressions and increase provider’s capacity to build and maintain emotional rapport with Black patients in provider-patient interactions. Specifically, as discussed above, the training focused on improving responding in key racially charged moments when biases were likely to be activated in providers (such as when patients describe past incidents of discrimination) and providers were at high risk for responding in ways that may be damaging to the provider-patient relationship.

The training protocol represented two potentially significant innovations over current mainstream anti-bias trainings. First, rather than directly focusing on changing implicit bias, the intervention focused on teaching skills related to mindfulness and acceptance. Mindfulness was defined as a psychological skill that can be learned: “Paying attention in a particular way: on purpose, in the present moment, and nonjudgmentally,” ([37], p. 4) with attention typically but not exclusively focused on one’s private experiences (i.e., feelings, thoughts, sensations). Likewise, acceptance was defined as “actively contacting psychological experiences — directly, fully, and without needless defense ” ([38], p. 1163). Evidence is accumulating that brief mindfulness skills trainings—which may include psycho-education about mindfulness, secular meditative practice to improve mindful awareness of ongoing thoughts and feelings (included biased thoughts and feelings), and group interactions—are effective at reducing bias in healthcare professionals [39]. In the current intervention, after an introductory didactic on racism and bias in medical care [40], rather than directly trying to reduce or suppress interracial anxieties and stereotypes that may arise in racially charged interactions, providers were taught mindfulness and acceptance skills to become more aware of these processes while reducing their functional impact on behavior. These exercises specifically were adapted from Acceptance and Commitment Therapy (ACT) [41], a contextual-behavioral science (CBS) intervention that employs mindfulness and acceptance processes to increase behavioral flexibility which has been shown to be effective across a variety of clinical disorders and treatment contexts [42, 43].

Second, the current intervention employed an innovative form of intergroup contact, which posits that, under certain conditions, contact between groups promotes positive attitudes and reduces prejudice between those groups [44]. Research documents that intergroup contact is effective and this is likely because contact facilitates closeness, which in turn improves empathy and perspective taking [45]. Thus, to maximize the benefits of contact, the intervention instantiated contact in terms of the central premise of the dominant social psychological model of interpersonal closeness, the *Interpersonal Process Model* (IPM [46, 47]). Specifically, the central premise of the IPM is that close and trusting relationships are formed from repeated, dyadic processes in which one person engages in vulnerable self-disclosure and the other person engages in responsiveness, defined as conveying understanding, validation and caring to the vulnerable individual. As per research on the IPM, the process requires reciprocity: both members of the dyad must engage in vulnerable self-disclosure and both must respond well to each other’s disclosures for benefits to accrue [48]. This is found across multiple relationship types [49, 50] including interracial relationships [51–53].

With respect to provider-patient interactions, the IPM’s element of vulnerability is likely present for many patients, simply as a function of being a patient, and this likely is amplified for Black patients, who may enter the interaction with fears of being misunderstood [54] or becoming the target of prejudice [55], and concerns about stereotype threat [33]. Regarding providers, the implicit biases with which they enter the interaction, and the likelihood that these biases may be activated during the interaction, make it harder for them to respond well and more likely that they will engage in behaviors that are documented to be damaging to the Black patient’s trust, satisfaction, and treatment adherence, such as those outlined above. In other words, while White provider-Black patient interactions involve intergroup contact, the IPM clarifies that such contact will not likely lead to improvements in Black patients’ trust in their doctors unless the doctors respond well to the patients’ vulnerable presentations.

Furthermore, according to research on the IPM, to address biases and improve White provider behavior, standard diversity trainings (in which White participants simply listen and bear witness to vulnerable stories shared by participants of color) are unlikely to impact connection and improve provider behavior and empathic responsiveness. Instead, White participants must also disclose vulnerable information, and have participants of color respond with responsiveness, for behavioral improvements to occur in interactions.

Thus, in the current intervention, participants engaged in intergroup contact exercises involving reciprocal exchanges of vulnerable self-disclosure and responsiveness. These exercises were adapted from empirically supported experiential training techniques used in Functional Analytic Psychotherapy (FAP), a CBS intervention that specifically trains clinicians in responsiveness to patients’ vulnerable behaviors [56, 57], including applications for racially diverse clinician-patient dyads [58]. FAP training emphasizes structured experiential exercises that have been demonstrated to improve clinician’s responsiveness in several clinical trials [59–61]. In these exercises, participants continued to practice acceptance and mindfulness skills to defuse from anxieties and stereotypes that often arise while engaging in vulnerable self-disclosures, empathic listening and responsiveness in interracial interactions.

We conducted a pilot randomized controlled trial of the intervention’s effects on provider responsiveness and emotional rapport building behavior in simulated interracial patient encounters, in which standardized Black patients specifically disclosed racially charged information and presented in ways likely to trigger negative stereotypes and microaggressive responses in the provider. The sample comprised a group of medical students and early professionals, an important population to study as they are easy to access for bias reduction trainings and, compared to later career providers, may have had fewer negative interracial experiences which are known to entrench bias and make it harder to change [6, 62]. We hypothesized that, compared to control providers, providers who received the intervention would demonstrate improved responsiveness and emotional rapport-building behaviors with the standardized patients. Although we did not generate specific hypotheses, in exploratory analyses we investigated differential effects of the intervention on White and non-White providers.

## Methods

### Trial design

**Fig. 1:**
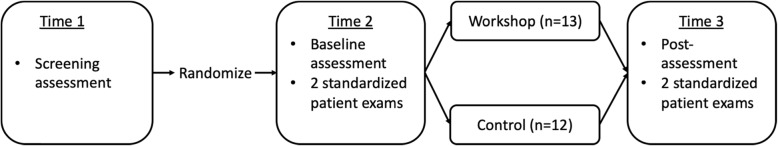
Trial Design

See Fig. 1 for trial design. Utilizing a randomized controlled parallel trial design, participants were assigned to either the intervention or no-intervention waitlist control condition, blocking by race so that there were equal numbers of Whites in each condition (approximately 60%; see Table 1), and equal numbers of non-Whites in each condition (approximately 40%). Randomization, including sequence generation and allocation, was performed using QMinim, a minimization application available online (https://sourceforge.net/projects/qminim/, [63]). The 3rd author performed all randomization procedures using this QMinim platform. Participants provided informed consent prior to participation, and the institutional review board approved the study. All participants completed questionnaires at screening (T1) and baseline (T2) and undertook two baseline standardized patient interactions (T2). The next day, participants either received the workshop (*n* = 13) or received no intervention (*n* = 12). Two clinical faculty administered the intervention jointly, a White male (JWK) and a Black female (MTW). Two days after the workshop, all participants conducted two more standardized patient interactions and completed post-experiment questionnaires (T3). Eligibility criteria initially included being a 3rd or 4th year medical student; this was broadened after recruitment began to include recent graduates and second-year students, increasing our sample size. No other changes were made after trial commencement. Recruitment began in May, 2016, and the study ran from September 17–September 21, 2016. The trial ended once all participants completed study procedures.

**Table 1:** Demographics by group

	Intervention providers	Control providers
Age: Mean (SD)	30.5 (5.16)	29.3 (6.33)
Gender: No. (%)		
Female	9 (69.2)	9 (75.0)
Male	4 (30.8)	3 (25.0)
Race: No. (%)		
Black	1 (7.7)	0 (0.0)
Asian	2 (15.4)	1 (8.3)
White	8 (61.5)	7 (58.3)
Other	2 (15.4)	4 (33.3)
Ethnicity: No. (%)		
Hispanic	2 (15.4)	10 (83.3)
Non-Hispanic	11 (84.6)	2 (16.7)

### Participants

The study was conducted at a naturopathic medical school in the [Pacific Northwest] region of the United States. Participants were medical students (84%) and recent graduates (16%), all of whom had been trained in and had previously delivered or were currently delivering primary care services at the University’s outpatient teaching clinic, where the study was conducted. Participants were paid $100 for completing the study. Recruitment materials described a study of cultural competency and occurred through announcements on student listservs and in classes and word-of-mouth; involvement was voluntary and extra-curricular. A total of 40 participants responded to recruitment materials. Of these, 15 were unable to commit to the study dates, leaving 25 participants who were randomized to condition. Thus, sample size was determined based on availability.

All randomized participants completed the study fully. Most participants were female (72%) and White (52%). The remainder were mixed race (Asian and White-20%; Black and White-4%), Asian (12%), Latino (8%), and Black (4%). Participants ranged from 23 to 45 years old, with most between 23 and 31 (72%). Most were single (60%). There were no significant differences in baseline characteristics by condition (see Table 1).

### Workshop intervention

The workshop (Table 2) took place in September 2016, as little as 2 days, and as much as 4 months, after participants completed prescreening. The workshop opened with an introduction and didactic on health disparities, stereotypes, microaggressions, interracial provider-patient interactions and racism [40]. Then, adapted from ACT, a guided, interracial eye-contact mindfulness exercise was performed to increase providers’ awareness and mindful acceptance of subtle expressions of bias that occur in interracial interactions. Then, in small, mixed-race groups, participants practiced the above mindfulness skills while reciprocally sharing and responding with empathy to each other’s personal life histories and personal narratives of loss and/or betrayal. These exercises were modified from exercises previously used to train therapists in responsiveness in FAP trainings (e.g. [60]). Finally, consistent with reviews of patient-centered communication education strategies that emphasize the value of practicing with specific examples [64] and the effectiveness of behavioral rehearsal as a skills training strategy [65], the intervention ended with an explicit practice component, in which participants broke into groups and practiced and received feedback on skills in role-play scenarios. A full description of the theoretical model and workshop techniques is described elsewhere [66].

**Table 2:** Workshop components

Component	Content	Duration
Introduction	• Presenter introductions and informed consent	30 min
Didactics	• Overview of medical health disparities • Research on interracial provider-patient interactions • Stereotypes and microaggressions	60 min
Mindfulness	• Interracial eye contact exercises to practice mindful awareness of attention, anxiety, and stereotypes	60 min
Contact	• Sharing of life histories and personal narratives of loss and betrayal in small interracial groups	120 min
Practice	• Role-play responding to patient prompts in small groups with modeling and feedback from presenters	60 min

### Standardized patient interactions

Four standardized patient interactions were developed to assess our primary hypotheses, with each patient presenting a specific *racial challenge* to the participants. Each standardized patient was played by a Black actor who was hired for the purposes of this study. Two of these actors were experienced standardized patients and two were new to this role; all received specific group training and practice with an experienced standardized patient coordinator at a major teaching hospital. For each standardized patient, the racial challenge instructions were scripted, but otherwise standardized patients were not scripted; they adhered to detailed case descriptions, including specific information about their condition and responses to likely questions from the provider.

The first scenario depicted a mid − 30’s, low-income woman with five children who requested assistance with fertility issues to have another child. This situation was chosen to elicit negative stereotypes of “welfare mothers,” potentially challenging the provider’s provision of emotional rapport by triggering paternalistic, controlling attitudes toward Black women’s fertility [67]. The second scenario depicted a mid-40’s man seeking medication to relieve chronic pain from a work accident, which may challenge rapport by eliciting stereotypes about Black people being drug abusers and medical misconceptions about having higher pain tolerances than Whites [68]. The third scenario depicted a mid-50’s man with poorly controlled diabetes, who reported that his last doctor told him he was “fat, Black, and lazy,” potentially eliciting documented stereotypes about compliance with diabetes care and decreasing provider’s likelihood of engaging in shared decision-making [69]. The fourth scenario depicted an early− 20’s woman with depression, a presentation that may elicit provider biases which lead to discounting depression in favor of more externalizing and antisocial conditions [70]. This patient complained that she could not get an appointment with a mental health provider but “a White girl would have gotten a callback,” a documented disparity in treatment access [71].

Participants interacted with two standardized patients per day at T2 (pre-intervention) and T3 (post-intervention). Standardized patients were randomly selected and blinded to participant condition. No participant interacted with the same patient twice. All participants were required to conduct a 10-min primary care encounter with each patient. The encounters occurred in a typical primary care exam room at a medical clinic associated with the medical school. A research assistant escorted participants to exam rooms where each standardized patient was waiting, and the research assistant knocked on the door to end the exam after exactly 10 min, such that only the participant and standardized patient were in the room for the exam. Before and after each exam, the standardized patient started and stopped the video-recordings of each exam with a fixed video-camera in the corner of each room. Participants were instructed not to talk with each other about the nature of their patients and—other than during the workshop (for workshop participants)—had no opportunities for interaction with each other during the study period.

### Observer-rated outcomes

Two sets of independent assessors, blinded to condition, rated each video-recorded standardized patient interaction. One set of two trained assessors evaluated overall emotional rapport-building, and another set of four trained assessors examined microaggressions and responsiveness specifically to the presentation of the racial challenge.

#### Emotional rapport building

We used the Roter Interactional Analysis System (RIAS), a turn-by-turn coding system in which each provider and patient utterance is coded into one of 40 categories of speech, which can be combined to create various summary scores. RIAS codes have demonstrated validity with a variety of samples and medical contexts in the US and abroad [16], including use with standardized patient interactions [72]. For the current study, two trained RIAS coders provided ratings, and both coders coded a subset of tapes (10%) for reliability (*r* = 0.92). Because standardized patients in our study were following a protocol and not responding naturally, we analyzed only provider codes, specifically the Emotional Rapport Building summary score which is a composite of doctor codes including emotional statements, legitimizing statements, concern statements, partnership statements, self-disclosure statements, and reassurance statements.

#### Responsiveness to racial challenges

We modified observer-based responsiveness coding systems used in previous IPM research [73, 74] that operationalized positive responsiveness in dyads as concrete instances of understanding, validation, and caring on a 0 to 3 scale. We expanded the previous 0 code, which originally combined both no responses (e.g., simply ignoring the challenge) and invalidating responses (e.g., defensive or microaggressive responses), into a negative responsiveness dimension and provided anchors and examples for each score, producing a Likert scale from – 3 to + 3, as shown in Table 3. Four coders were trained to high reliability before beginning study coding, evaluated with 10 practice tapes created by the authors (Interclass correlation coefficient—*ICC* > 0.98). A subset of study tapes (10%) was coded by all four raters (*ICC* = 0.73).

**Table 3:** Responsiveness to racial challenges scale with scoring examples

Scenario	Score and Anchor						
	-3: Strong, immediate negative reaction	-2: Strong negative reaction anytime	−1: Mild negative reaction/ ignoring	0: Passive reaction	1: Mild positive reaction	2: Strong positive reaction anytime	3: Strong, immediate positive reaction
Male (60s) with diabetes: “My last doctor said I was, ‘fat, black, and lazy’.”	-What were you doing to make her say that? -A lot of black men have trouble motivating themselves to take care of their health … -I’m sure they didn’t mean that.		-Okay, and what are your blood pressure levels?	-Fat, black, and lazy, I bet you didn’t like that. -That’s an odd way to talk to a patient.	-Yikes. -That’s not okay! -That must have been painful.	-I’m sorry you experienced that—it sounds awful. -Some doctors are just idiots. -I just can’t believe … I mean I believe it happened, but I can’t believe a doctor said that.	
Female (20s) with depression and anxiety: “A white girl would have gotten more callbacks.”	-What did you say on the phone? -I’m sure it wasn’t a race thing. -Y’know, people get so busy this time of year, they probably just didn’t have openings. -I don’t think anyone would base acceptance on race.		-So what kinds of symptoms are we talking about?	-You think not getting a call back was based on your race.	-It must have been hard not to get a call back!	-That sounds really frustrating. I get that you have these experiences all the time, you’re right that white girls get callbacks; I won’t deny racism with you. - I take this seriously and you’re safe to talk about this with me.	
Female (late 30s) with five children asking for fertility treatment	-How many kids do you have? (with judgmental inflection) -You could have a basketball team.		-Have you always wanted such a huge family? -Great, welcome! We’ll see how we can help you!	-That sounds like a bad experience. -It sounds like your provider didn’t support your values.	-I apologize that you had to go through that … it’s not really our place to put in our opinion.	- I want to make sure we’re talking about anything we need to … that doesn’t sound right and that wouldn’t land right. -If I ever do that to you, we can talk about it, to make this the best experience for you.	
Male (40s) with chronic pain seeking pain medications: Bad ER experience	-We’re trained not to give pain meds to patients who present a risk for addiction.		-Why do you need these pain meds? -What kind of pain meds were you seeking?	-You felt profiled by ER doctors.	-The ER doctors are not there to assume, right? They’re there to listen to you.	-That sounds really invalidating and unfair. It sounds like you were treated like a criminal because of the color of your skin.	

#### Offensiveness, bias, recommendations, and patient experience

Responsiveness coders answered the following yes/no questions for each standardized patient interaction: 1) *Did the doctor say anything offensive?* 2) *Was the doctor overtly biased against the patient?* 3) *Would you recommend that a Black friend with the same problem as this patient see this doctor over any other doctor?* and 4) *Do you think the patient had a positive experience?* Questions 1 and 2 showed severe range restriction (11 and 1% “yes” responses, respectively) and were not analyzed. Questions 3 and 4 were dummy coded as “no” = 0 and “yes” = 1 and averaged across the two patient interactions at each time point. We found moderate interrater reliability across the raters for Question 3 (*ICC* = 0.593) and excellent reliability for Question 4 (*ICC =* 1.0).

### Participant self-reported outcomes

#### Ethnocultural empathy

At T2 and T3, participants completed the 30-item, Everyday Multicultural Competencies/Revised Scale of Ethnocultural Empathy (EMC/RSEE), which has strong evidence for factor structure, reliability and discriminant validity [75]. We hypothesized changes on the *Empathic Perspective-Taking* and *Acting as an Ally* subscales. The former demonstrated poor internal consistency (α = 0.45) and was not included in analyses. The *Acting as an Ally* subscale demonstrated good internal consistency (α‘s > .70 at both time points) after removing one item from the composite. The final items for the *Acting as an Ally* subscale are presented in Table 4.

**Table 4:** Acting as an Ally subscale items (bold items are reverse coded)

**I don’t care if people make racists statements against other racial or ethnic groups.** When I hear people make racist jokes, I tell them I am offended even though they are not referring to my racial or ethnic group. When I see people who come from a different racial or ethnic background succeed in the public arena, I share their pride. When I know my friends are treated unfairly because of their racial or ethnic backgrounds, I speak up for them.	

#### Attitudes toward various ethnic/racial groups

To assess explicit *attitudes towards various ethnic and racial groups*, at T1 and T3, participants provided feeling thermometer ratings for different groups of people, including African Americans and 13 other demographic categories, from 0 (*extremely unfavorable*) to 100 (*extremely favorable*). Low scores on feeling thermometers are interpreted as a simple indicator of explicit prejudice [76] and feeling thermometers demonstrate significant correlations with objective behavioral indicators of discrimination in meta-analyses [77] and propensity to commit subtle acts of racism [78]. There was strong internal consistency (αs > .94) at both time points when looking at all 15 demographic categories, so we computed a mean score indicating overall attitudes toward people of color.

#### Working alliance

After each standardized patient interaction at T2 and T3, participants completed the 12-item Bond subscale of the Working Alliance Inventory (WAI), a widely used measure to assess the provider-patient alliance in therapeutic interactions [79] which has been modified for medical settings, including with standardized patients [80]. Our sample showed good internal consistency in both interactions at both time points (α*s* > .85). The *Bond* subscale items are presented in Table 5.

**Table 5:** Bond subscale items (bold items are reverse coded)

**I feel uncomfortable with this patient** I feel I really understand this patient I believe this patient likes me I am genuinely concerned for this patient’s welfare This patient and I respect each other **I feel that I am not totally honest about my feelings toward this patient** I am confident in my ability to help this patient I appreciate this patient as a person This patient and I have built a mutual trust Our relationship is important to this patient **This patient has some fears that if she/he says or does the wrong things, I will stop working with him/her** I respect this patient even when he/she does things that I do not approve of	

#### Interaction closeness

After each standardized patient interaction at T2 and T3, participants answered three questions about their experience of the interaction. First, they completed the *Inclusion of the Other in the Self* (IOS) scale, a well-validated, single-item, pictorial measure of relational closeness [81, 82] which has been used to assess closeness in interracial contexts [83] and between patients and providers [84]. The other two closeness items were the following: 1) *Relative to all your other relationships with patients (outside of this study), how would you characterize your relationship with this patient?* and 2) *Relative to what you know about other patient’s relationships with their doctors, how would you characterize your relationship with this patient?* Agreement with these items was assessed on 7-point Likert scales ranging from 1 (*less close)* to 7 (*more close*). We computed a mean composite score of these two items (α > 0.67).

### Data analysis

For primary analyses, repeated measures analysis of variance (ANOVA) tests were used to assess patterns of change from T1/T2 to T3 by condition. Of interest were significant interactions between time and condition. Paired t-tests for participants within each condition were used to explore significant (*p* < .05) or approaching significant (*p* < .10) interactions. *Cohen’s d* was computed to characterize the size of these effects, using guidelines of small (> 0.2), medium (< 0.5) and large (> 0.8) [85]. Results were split by provider race for additional exploratory moderator analyses, using the same strategy.

## Results

### Preliminary analyses

There were no significant differences between groups at T1 or T2 on any observer-based or provider self-reported outcomes. There were no differences between observer-based ratings of the first and second simulated patient interviews within T2 or T3, so scores for the two T2 standardized patient interviews and the two T3 interviews were summarized into simple means. Descriptive statistics for all outcome variables by time and condition are reported in Table 6.

**Table 6:** Primary outcome variables by condition and time, with paired (pre-post) t-test results for each condition

Measure	Intervention Providers					Control Providers				
	Pre M (SD)	Post M (SD)	*t*	*P*	*Cohen’s d*	Pre M (SD)	Post M (SD)	*t*	*p*	*Cohen’s d*
Observer-rated										
Emotional rapport building	7.27 (4.66)	15.08 (4.69)	−5.39	.000	1.67	10.21 (4.84)	10.21 (3.05)	0.00	1.000	0.00
Response to racial challenges	0.08 (0.84)	1.65 (0.92)	−3.43	.005	1.78	0.71 (1.05)	0.74 (0.93)	−0.08	.942	0.03
Provider offensive?	0.19 (0.33)	0.04 (0.14)	–	–	–	0.15 (0.27)	0.13 (0.23)	–	–	–
Provider biased?	0.04 (0.14)	0.00 (0.00)	–	–	–	0.00 (0.00)	0.00 (0.00)	–	–	–
Recommend provider?	0.17 (0.24)	0.76 (0.33)	−5.05	.000	2.04	0.36 (0.36)	0.40 (0.31)	−0.26	.797	0.12
Patient positive experience?	0.42 (0.34)	0.87 (0.22)	−3.49	.004	1.57	0.52 (0.41)	0.63 (0.23)	−0.92	.376	0.33
Provider self-report										
Acting as an ally	4.12 (0.68)	4.20 (0.69)	−0.65	.530	0.12	4.32 (0.66)	4.23 (0.59)	1.80	.096	0.14
Attitudes towards minorities	60.28 (11.87)	77.03 (12.53)	−4.68	.001	1.37	66.81 (15.06)	69.07 (15.74)	−1.36	.200	0.15
WAI-Bond	5.63 (0.39)	6.09 (0.41)	−4.80	.000	1.15	5.90 (0.47)	6.01 (0.34)	1.21	.254	0.27
IOS	4.15 (1.61)	5.27 (1.28)	−3.93	.002	0.77	4.46 (1.50)	4.42 (1.14)	0.12	.908	0.03
Interaction closeness	4.58 (0.63)	5.58 (0.52)	−4.45	.001	1.73	5.04 (0.86)	5.06 (0.81)	−0.15	.886	0.02

*WAI* Working Alliance Inventory, *IOS* Inclusion of Other in the Self Scale

### Observer-based outcomes

For *emotional rapport building,* a significant interaction was found (*p* = .001, *η*^*2*^ = .40), with Intervention participants demonstrating a significant and large improvement from pre-intervention to post-intervention (*p* < .001, *Cohen’s d* = 1.67) and Control participants demonstrating no change (*p* = 1.00*, d* = 0.00). For *responsiveness to racial challenge*s, a significant interaction was found (*p* = .016*, η*^*2*^ = .23), with Intervention participants demonstrating a significant and large improvement from pre-intervention to post-intervention (*p* = .005*, d* = 1.78) and Control participants demonstrating no change (*p* = .942*, d* = 0.03). For *provider recommendations,* a significant interaction was found (*p* = .005, *η*^*2*^ = .29), with Intervention participants demonstrating a significant and large improvement from pre-intervention to post-intervention (*p* < .001*, d* = 2.04) and Control participants demonstrating no change (*p* = .797*, d* = 0.12). No significant interaction effect was found for observed-assessed *patient positive experiences* (*p* = .058, *η*^*2*^ = .15). Although the Intervention participants did demonstrate a significant and large improvement from pre-intervention to post-intervention (*p* = .004*, d* = 1.57), Control participants evidenced a small and non-significant improvement (*p* = .376*, d* = .33).

### Provider self-reported outcomes

For *acting as an ally,* no significant effects were found (*p* = .235, *η*^*2*^ = .06). For *attitudes towards minorities,* a significant interaction was found (*p* = .002, *η*^*2*^ = .36), with Intervention participants demonstrating a significant and large improvement from pre-intervention to post-intervention (*p* = .001*, d* = 1.37) and Control participants demonstrating a moderate and non-significant improvement (*p* = .200*, d* = 0.15). For *WAI-bond,* a significant interaction was found (*p* = .017, *η*^*2*^ = 0.22), with Intervention participants demonstrating a significant and large improvement from pre-intervention to post-intervention (*p* < .001*, d* = 1.15) and Control participants demonstrating a moderate and non-significant improvement (*p* = .254*, d* = 0.27). For *IOS,* a significant interaction was found (*p* = .017, *η*^*2*^ = .23), with Intervention participants demonstrating a significant and moderate-to-large improvement from pre-intervention to post-intervention (*p* = .002*, d* = .77) and Control participants demonstrating no change (*p* = .908*, d* = 0.03). For *interaction closeness,* a significant interaction was found (*p* = .001, *η*^*2*^ = .36), with Intervention participants demonstrating a significant and large improvement from pre-intervention to post-intervention (*p* = .001*, d* = 1.73) and Control participants demonstrating no change (*p* = .886*, d* = 0.02).

### Moderation of results by race

In exploratory analyses, we split the data by provider race and re-ran outcome analyses for the 13 White participants and the 12 participants who self-identified as Black, Asian, and other, who we refer to as participants of color (POC). For the White participants (Table 7), the observer-rated outcomes all remained significant. Specifically, the interaction between time and condition was significant for *emotional rapport building* (*p* = .002; *η*^*2*^ = .60), with White Intervention participants demonstrating a significant and large improvement from pre-intervention to post-intervention (*p* = .001*, d* = 2.40) and White Control participants demonstrating no change (*p* = .761*, d* = 0.16). For *responsiveness to racial challenges*, the interaction was significant (*p* = .001; *η*^*2*^ = .62), with White Intervention participants demonstrating a significant and large improvement from pre-intervention to post-intervention (*p* = .011*, d* = 2.53) and White Control participants demonstrating a significant deterioration (*p* = .019*, d* = 0.87). For observer-based *recommendations,* the interaction was significant (*p* < .001, *η*^*2*^ = .76), with White Intervention participants demonstrating a significant and large improvement from pre-intervention to post-intervention (*p* = .000*, d* = 4.52) and White Control participants demonstrating no change (*p* = .110*, d* = 1.13). For observer-based *patient experiences,* the interaction was significant (*p* = .03, *η*^*2*^ = .35), with White Intervention participants demonstrating a significant and large improvement from pre-intervention to post-intervention (*p* = .004*, d* = 2.38) and White Control participants demonstrating no change (*p* = .849*, d* = 0.13).

**Table 7:** Primary outcome variables for White participants by condition and time, with paired (pre-post) t-test results for each condition

Measure	Intervention Providers					Control Providers				
	Pre M (SD)	Post M (SD)	*t*	*p*	*Cohen’s d*	Pre M (SD)	Post M (SD)	*t*	*p*	*Cohen’s d*
Observer-rated										
Emotional rapport building	6.79 (4.50)	17.29 (4.25)	−6.43	.001	2.40	10.83 (6.26)	10.08 (2.18)	0.32	.761	0.16
Response to racial challenges	−0.36 (0.75)	1.86 (0.99)	−3.65	.011	2.53	1.13 (0.95)	0.42 (0.66)	3.40	.019	0.87
Provider offensive?	0.21 (0.39)	0.07 (0.19)	–	–	–	0.17 (0.26)	0.08 (0.20)	–	–	–
Provider biased?	0.00 (0.00)	0.00 (0.00)	–	–	–	0.00 (0.00)	0.00 (0.00)	–	–	–
Recommend provider?	0.04 (0.09)	0.86 (0.24)	−6.94	.000	4.52	0.60 (0.42)	0.21 (0.25)	1.94	.110	1.13
Patient positive experience?	0.36 (0.38)	1.00 (0.00)	−4.50	.004	2.38	0.54 (0.40)	0.58 (0.20)	−0.20	.849	0.13
Provider self-report										
Acting as an ally	4.20 (0.54)	4.23 (0.48)	−0.14	.895	0.06	4.40 (0.46)	4.30 (0.47)	2.24	.076	0.22
Attitudes towards minorities	61.20 (13.97)	78.58 (13.07)	−2.86	.029	1.28	73.23 (10.88)	77.79 (11.53)	−3.26	.023	0.31
WAI-Bond	5.49 (0.30)	6.07 (0.35)	−7.04	.000	1.78	5.91 (0.57)	5.97 (0.39)	−.40	.706	0.12
IOS	3.79 (1.55)	4.93 (1.13)	−2.83	.030	0.84	4.67 (1.78)	5.00 (1.10)	−.88	.421	0.22
Interaction closeness	4.10 (1.13)	5.32 (0.43)	−3.00	.024	1.43	5.42 (0.66)	5.29 (0.90)	.89	.415	0.16

*WAI* Working Alliance Inventory, *IOS* Inclusion of Other in the Self Scale

Three of the four significant self-reported outcomes also remained significant for the White providers. No significant interaction was found for *attitudes towards minorities* (*p* = .08, *η*^*2*^ = .25), but in follow-up tests White Intervention participants demonstrated a significant and large improvement from pre-intervention to post-intervention (*p* = .029*, d* = 1.28) and White Control participants demonstrated a significant moderate improvement (*p* = .023*, d* = 0.31). For *WAI: Bond,* the interaction was significant (*p =* .01, *η*^*2*^ = .46), with White Intervention participants demonstrating a significant and large improvement from pre-intervention to post-intervention (*p* < .001*, d* = 1.78) and White Control participants demonstrating no change (*p* = .706*, d* = 0.12). For provider self-reported *interaction closeness,* the interaction was significant (*p* = .014, *η*^*2*^ = .44), with White Intervention participants demonstrating a significant and large improvement from pre-intervention to post-intervention (*p* = .024*, d* = 1.43) and White Control participants demonstrating no change (*p* = .415*, d* = 0.16). The interactions were not significant for *Acting as an Ally* (*p* = .585, *η*^*2*^ = .03) and *IOS* (*p* = .177, *η*^*2*^ = .16).

For the 12 participants of color (Table 8), all but one of the effects were in the same direction as the larger set of effects but were largely non-significant. For observer-rated outcomes, there were no significant findings, including *emotional rapport building* (*p* = .149, *η*^*2*^ = .20), *response to racial challenges* (*p* = .937, *η*^*2*^ = .01), *recommendations* (*p* = .831, *η*^*2*^ = .01), or *patient experiences* (*p* = .809, *η*^*2*^ = .01). For self-reported outcomes, a significant interaction was found for *attitudes towards minorities* (*p* = .007, *η*^*2*^ = .53) with POC Intervention participants demonstrating a significant and large improvement from pre-intervention to post-intervention (*p* = .008*, d* = 1.39) and POC Control participants demonstrating no change (*p* = .992*, d* = 0.00). No significant effects were found for *WAI-bond* (*p* = .398, *η*^*2*^ = .07) or *interaction closeness* (*p* = .054, *η*^*2*^ = .32), although for *interaction closeness* in follow-up tests POC Intervention participants demonstrated a significant and large improvement from pre-intervention to post-intervention (*p* = .027*, d* = 2.08) and POC Control participants demonstrated no change (*p* = .530*, d* = 0.20). There were no significant effects observed for POC participants for *acting as an ally* (*p* = .183, *η*^*2*^ = .17) or *IOS* (*p* = .066, *η*^*2*^ = .30).

**Table 8:** Primary outcome variables for participants of color by condition and time, with paired (pre-post) t-test results for each condition

Measure	Intervention Providers					Control Providers				
	Pre M (SD)	Post M (SD)	*t*	*p*	*Cohen’s d*	Pre M (SD)	Post M (SD)	*t*	*p*	*Cohen’s d*
Observer-rated										
Emotional rapport building	7.83 (5.21)	12.50 (4.05)	−2.49	.056	1.00	9.58 (3.38)	10.33 (3.96)	−0.45	.670	0.20
Response to racial challenges	0.58 (0.66)	1.42 (0.86)	−1.36	.233	1.10	0.29 (1.05)	1.06 (1.10)	−1.29	.253	0.72
Provider offensive?	0.17 (0.26)	0.00 (0.00)	–	–	–	0.13 (0.31)	0.17 (0.26)	–	–	–
Provider biased?	0.08 (0.20)	0.00 (0.00)	–	–	–	0.00 (0.00)	0.00 (0.00)	–	–	–
Recommend provider?	0.33 (0.26)	0.65 (0.40)	−2.05	.095	0.95	0.22 (0.24)	0.58 (0.26)	−2.66	.045	1.44
Patient positive experience?	0.50 (0.32)	0.72 (0.25)	−1.14	.308	0.77	0.50 (0.45)	0.67 (0.26)	−1.58	.175	0.46
Provider self-report										
Acting as an ally	4.03 (0.85)	4.17 (0.93)	−1.20	.286	0.16	4.23 (0.86)	4.17 (0.73)	0.79	.465	0.08
Attitudes towards minorities	59.21 (10.06)	75.22 (12.83)	−4.20	.008	1.39	60.38 (16.77)	60.35 (15.14)	0.01	.992	0.00
WAI-Bond	5.79 (0.45)	6.13 (0.51)	−1.84	.126	0.71	5.88 (0.41)	6.05 (0.31)	−1.39	.223	0.47
IOS	4.58 (1.72)	5.67 (1.44)	−2.49	.056	0.69	4.25 (1.29)	3.83 (0.93)	0.71	.507	0.37
Interaction closeness	4.75 (0.59)	5.88 (0.49)	−3.09	.027	2.08	4.67 (0.92)	4.83 (0.70)	−0.67	.530	0.20

*WAI* Working Alliance Inventory, *IOS* Inclusion of Other in the Self Scale

## Discussion

This pilot study provides initial support for the efficacy of a workshop intervention that targeted decreased provider microaggressive responding and improved provider responsiveness and emotional rapport, including expressions of empathy and perspective taking, in key racially charged moments in interracial provider-patient interactions. With respect to our primary observer-based outcomes, two independent, blind teams of coders observed significant behavioral improvements for intervention providers but not control providers on provider responsiveness and emotional rapport in simulated interracial patient encounters with standardized Black patients. The *emotional rapport building* finding suggests that over the course of two 10-min simulated patient interviews with Black patients, intervention providers demonstrated greater improvements in building emotional rapport, evidenced by their increased use of statements of concern, validation, and empathy. The *responsiveness* finding suggests that the intervention providers were more likely to specifically respond well to the patients in racially charged moments when providers are likely to struggle and engage in behaviors experienced as microaggressive by patients. Although emotional rapport and responsiveness are not often the focus of bias-reduction interventions, they have demonstrated relationships to patients’ trust in and feeling respected by their doctors, visit satisfaction, and experiences of patient-centered care [10, 13, 14, 18], which are thought of as key mediators of a fundamental pathway through which provider implicit biases affect racial health disparities in both quality and outcomes of medical care [86, 87]. Along these lines, coders also reliably reported an increased likelihood of recommending intervention providers but not control providers to other Black patients.

With respect to self-reported outcomes, the intervention providers themselves, compared to control providers, reported improved explicit attitudes toward ethnic and racial minorities and perceptions of improved working alliances and closeness with the specific post-intervention Black standardized patients with whom they interacted. Exploratory analyses of results for White participants and participants of color suggested that for Whites, intervention participants demonstrated larger and more robust changes compared to control participants, while for POCs, intervention participants’ improvements compared to control participants’ were smaller and not often significant. We note, however, the effect sizes for POC intervention improvements were often still large. For example, our measure of attitudes towards minorities, which is a composite that encapsulates attitudes towards a variety of marginalized identities, showed a large and significant change for POC intervention participants. Likewise, large, albeit non-significant, effects were observed for POC intervention participants for the two primary observer-based measures of provider behavior in the simulated patient interactions. These analyses by participant race, however, were exploratory and should not be over-interpreted. A conservative conclusion is that there is no evidence that the intervention is harmful or problematic for participants of color and future research is warranted to explore its benefits.

At the conceptual level, although the IPM likely is the most well-studied social psychological model of how close, intimate relationships develop in dyads, to our knowledge the current report is the first application of key premises of this model in the context of provider-patient interracial relationships. This premise, emphasizing the importance of reciprocal, vulnerable exchanges between individuals, was a central feature of the workshop. Thus it may be suggested that it had the hypothesized impact on provider empathy. That said, our research design tested a multi-component workshop (including didactics, mindfulness training, vulnerable contact, and behavioral rehearsal) and we have no evidence for which components of the workshop were most important in producing observed effects. The current findings encourage more precise applications and evaluations of the IPM in this context as well as the other workshop components in combination and isolation.

### Limitations

A primary limitation of the current report is that the sample size is small and restricted to medical students and young professionals in a specific training setting. Regarding sample size, our confidence in the robustness of our results is bolstered by the fact that results are consistent across multiple measures, including results obtained from the participants themselves and two sets of blind, independent behavioral coders. Regarding the sample composition, the intervention was designed to target normative social psychological processes of bias that are conclusively operative for most White individuals, regardless of professional orientation, and thus are likely expressed across many medical contexts [88–90], and there is no reason to believe that the current sample is unique in its susceptibility to bias or responsiveness to the intervention. That said, future research evaluations of the intervention model with additional, larger and more diverse groups of medical students and professionals are warranted.

Second, our assessment-only, wait-list control condition allowed us to control for the effects of our assessment procedures but offered no meaningful control over non-specific intervention effects or comparison to other bias-reduction strategies. Furthermore, the expectation of a future workshop may have influenced our control participants’ responses in unknown ways. For example, our secondary, self-report outcomes may have been biased in favor of workshop participants and against control participants who knew they did not receive the intervention. Future research should include more active comparison conditions for a more robust test of our intervention and its proposed mechanisms.

An additional limitation is that the current study provides evidence for immediate behavioral improvements following the intervention but no follow-up assessments were conducted. While immediate behavior change is important, and may be seen as the first step in a sustainable behavior change agenda, it is likely that additional follow-up or supplemental interventions are necessary for long-term change. It is also possible that the effects are sustainable, especially because the intervention emphasized active learning, practice, and feedback on concrete skills, including role-plays in realistic situations, as per recommendations from dissemination and implementation science [91, 92]. To our knowledge, these recommended intervention practices have not previously been tested in this setting. Future research with longer-term follow-up, and development of supplemental follow-up interventions, is recommended.

Finally, we note that the use of standardized patients is a strength and a limitation. An important strength is that changes in participant behavior, across multiple measures, were observed by independent coders, as actual behavior change in medical provider-patient interactions is the ultimate intervention goal. For example, studies that do show changes in implicit bias often show no corresponding behavioral changes, calling into question the value of focusing solely on implicit measures [35]. Furthermore, there is evidence that simulated patient training is important and generalizable across multiple training contexts [93, 94]. Standardized patients are also important tools for dissemination and implementation research outcome assessments, as the control provided by standardized patients allows for efficient and strategic testing of key skills in response to patient presentations that are unpredictable and variable in actual encounters [95]. That said, one limitation is that simply repeated exposure to the Black standardized patients, each presenting a different racial challenge, may have resulted in expectancy effects, reduction in tension and improved performance from Time 2 to Time 3 [96]. However, data from our control participants – who evidenced no changes in behavioral performance from Time 2 to Time 3 while experiencing the same standardized patients – mitigates this concern. Nonetheless, future research on the current training model should incorporate observation of actual patient interactions and outcomes for a full test of the proposed intervention and documentation of its effects on racial health disparities in medical care. It is likely also important to examine the effects of the intervention on implicit bias; findings that the intervention does not change implicit bias may indicate that change in implicit bias is not needed for important behavioral outcomes to change. In contrast, if the intervention changes implicit bias, measuring outcomes of subsequent iterations of the intervention will be easier, as implicit bias is significantly easier and quicker to measure than verbal and non-verbal behavior in real interactions.

## Conclusion

We present an innovative intervention to improve provider treatment of Black patients. This intervention includes elements informed by mindfulness and acceptance and the IPM of intimacy (i.e. interracial communication characterized by vulnerability and responsiveness). To our knowledge, this report constitutes the first evidence for the benefits of such an approach for interracial doctor-patient interactions. The intervention improved provider beliefs and behaviors across a range of measures, particularly for White providers. Because the intervention is modularized, it can easily be incorporated into ongoing medical education curricula, such as weekly didactics. That said, the current sample size and limitations prevent the drawing of definitive conclusions. Our preliminary evidence suggests that, with additional evaluation, this intervention strategy could be effective for addressing disparities in patient care that have downstream effects on patient health outcomes.

## Data Availability

The data generated during the current study are not publicly available due to individual privacy issues (e.g., videotapes), but data will be shared by the corresponding author on reasonable request.

## Abbreviations

CBS: Contextual-behavioral sciences
ACT: Acceptance and commitment therapy
FAP: Functional analytic therapy
RIAS: Roter interactional analysis system
EMC/RSEE: Everyday Multicultural Competencies/Revised Scale of Ethnocultural Empathy
WAI: Working Alliance Inventory
IOS: Inclusion of the Other in the Self
ANOVA: Analysis of variance
ICC: Interclass correlation coefficient
IPM: Interpersonal process model
